# Red Blood Cell Glycation Triggers In Vivo Cerebral Erythrophagocytosis in Adult Zebrafish in a Model Mimicking Hemorrhagic Stroke

**DOI:** 10.1002/cph4.70088

**Published:** 2026-01-06

**Authors:** Elena Morello, Philippe Rondeau, François Canonne‐Hergaux, Emmanuel Bourdon, Matthieu Bringart, Olivier Meilhac, David Couret, Nicolas Diotel

**Affiliations:** ^1^ Université de la Réunion, INSERM, UMR 1188 Diabète Athérothrombose Thérapies Réunion Océan Indien (DéTROI) Saint‐Pierre France; ^2^ CHU de La Réunion Saint‐Pierre France

**Keywords:** animal model, erythrocytes, glycation, intracerebral hemorrhage, neuro‐inflammation/microgliosis, zebrafish

## Abstract

Hemorrhagic stroke, particularly intracerebral hemorrhage (ICH), is a highly lethal subtype of stroke, responsible for a poor prognosis and a high rate of disability. ICH is characterized by the extravasation of red blood cells (RBCs) into the central nervous system. Efficient clearance of RBC lysis products is critical. Erythrophagocytosis may serve as a key protective mechanism in mitigating secondary damage of ICH. Interestingly, diabetes is known to promote RBC glycation and to exacerbate the consequences of ICH. However, the link between glycation, RBC clearance and neurological outcomes after ICH is not clear. In this study, we aimed to decipher the role of glycated RBCs in ICH. For this, we used human RBCs glycated by methylglyoxal (MGO), an intermediate product of glycolysis recognized as one of the most potent glycating agents in humans. These glycated RBCs displayed altered morphology, reduced deformability capacity and increased eryptosis. We then proceeded to RBC microinjection into the brain parenchyma of *Tg(mpeg1.1:mCherry)* zebrafish allowing the visualization of both microglia and macrophages. Although the presence of RBCs in the parenchyma did not affect the increased recruitment of mpeg1.1‐positive cells to the injection site compared to vehicle injection, RBC glycation significantly enhanced erythrophagocytosis. In conclusion, we established an innovative model of ICH, and we demonstrated enhanced RBC phagocytosis in the brain under glycated conditions. Further research is needed to understand the consequence of such process in ICH damage. Finally, our model may contribute to the identification of specific neurorestorative therapeutics aimed at improving brain plasticity after stroke in diabetic context.

## Graphical abstract

In zebrafish, glycated RBCs promoted increased erythrophagocytic activity in microglia/macrophages, revealing a potential mechanism linking diabetes‐induced RBC glycation to exacerbated inflammatory responses and secondary damage after ICH.

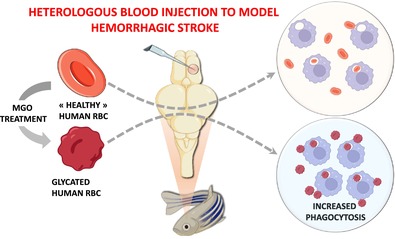

## 1 Introduction

Stroke represents the leading cause of acquired disabilities worldwide and the second cause of mortality. It can be classified into two main subtypes: ischemic and hemorrhagic strokes. Although the hemorrhagic subtype accounts for only 18%–28% of cases, it is far more lethal than the ischemic one (50% first‐year mortality rate vs. 28% for ischemic stroke) (Feigin et al. 2022). Hemorrhagic stroke is also associated with a higher risk of acquired disability (Li et al. 2022; Feigin et al. 2025), with 70% of survivors having functional disability and a poor prognosis (Haupenthal et al. 2021). Together, these data highlight the fact that hemorrhagic stroke represents a determinant public health issue.

Hemorrhagic stroke results from the non‐traumatic rupture of an intra‐cranial blood vessel, and corresponds mainly to intracerebral hemorrhage (ICH), which accounts for 28% of all incident stroke subtypes (Feigin et al. 2025). ICH can be either primary (spontaneous) or secondary to other conditions (i.e., neoplasm, arteriovenous malformation notably) (Aronowski and Zhao 2011; De Morais Filho et al. 2021). ICH is characterized by a primary brain lesion with the formation of a hematoma/edema associated with disrupted blood–brain barrier (BBB) and followed by a secondary brain lesion. This secondary brain lesion involves the toxicity of blood components, particularly the erythrocytes (Puy et al. 2023). In this article, we focus on the hemorrhage resolution phase: phagocytic cell recruitment and erythrophagocytosis. The presence of red blood cells (RBCs) in the brain parenchyma may be linked not only to a hemorrhagic stroke, but also to the hemorrhagic transformation of an ischemic stroke, which also affects many patients, particularly after recanalization. Hemorrhagic transformation is also increased in diabetic conditions (Grisotto et al. 2021).

Indeed, hemoglobin from RBCs generates oxidative stress in the brain parenchyma and induces a pro‐inflammatory response activating microglia and peripheral macrophages (Aronowski and Zhao 2011; Bulters et al. 2018; Tschoe et al. 2020). The scavenging of RBCs by these phagocytic cells is a crucial process in modulating the extent of neuronal damage.

It is noteworthy that diabetes and hyperglycemia have been identified as risk factors for the severity of ICH, these conditions being associated with an increased risk of mortality and poor recovery (Caceres and Goldstein 2012; Tapia‐Pérez et al. 2014; De Morais Filho et al. 2021). In such hyperglycemic conditions, RBCs are subject to glycation, resulting in the accumulation of advanced glycation end products (AGEs). This process involves methylglyoxal (MGO), an intermediate product of glycolysis, which is one of the most glycating agents in humans (Schalkwijk and Stehouwer 2020; Tupe et al. 2020; Turpin et al. 2021) and is commonly observed in individuals with diabetes (Schalkwijk and Stehouwer 2020). The glycation of RBCs is responsible for alterations of RBC structure and function (Babu and Singh 2004). It is suggested that such glycation processes could also impact RBC phagocytosis and consequently impair the classical steps of brain repair (Catan et al. 2019). Besides, the role of microglia following ICH remains misunderstood, particularly in diabetic/hyperglycemic conditions.

Zebrafish is an interesting model for investigating brain plasticity, largely due to its remarkable capacity for regenerating large cerebral lesions (März et al. 2011; Kizil et al. 2012; Schmidt et al. 2014; Diotel et al. 2020; Ghaddar, Lübke, et al. 2021). Zebrafish ICH models are currently being used during development, with the aim of inducing spontaneous brain hemorrhages (Crilly et al. 2018). However, despite the interest of such existing models in elucidating the mechanisms underlying the onset of ICH during development, there are certain concerns about their translation to the adult. So far, the use of adult zebrafish to investigate the influence of RBC presence within the parenchyma on neuroinflammatory processes, particularly microgliosis, is a promising avenue of research.

The aims of this study were firstly to set up a model of RBC MGO‐mediated glycation, then to establish a fast and efficient ICH model in adult zebrafish by local microinjection of RBCs into the telencephalon and finally to investigate the impact of RBC glycation on the recruitment of phagocytic cells (microglia and macrophages) and on erythrophagocytosis. Indeed, we hypothesize that RBC glycation could disrupt immune cell recruitment and their phagocytic capacity during brain regeneration.

## 2 Material and Methods

### 2.1 Red Blood Cell (RBC) Preparation

Blood samples from healthy human donors (three to five) obtained from the French blood national agency (EFS‐LR agreement number #2018001378) were collected in EDTA tubes (BD vacutainer). Blood was transferred to 15 mL tubes and centrifuged at 2000 g for 15 min. The supernatant was removed and replaced with an equivalent volume of 0.9% NaCl. This washing process was repeated three times, and RBCs were finally resuspended in PBS (Phosphate Buffered Saline, an isotonic buffer containing 140 mM NaCl, 2.7 mM KCl, 10 mM PO_4_^3−^, pH 7.4) at the targeted hematocrit (30%).

### 2.2 Fluorescent RBC Labelling Intended for Injection in Zebrafish Telencephalon

The PKH67 Green Fluorescent Linker kit (Sigma‐Aldrich, Saint‐Louis, MO) was used as previously described (Turpin, Apalama, et al. 2022). Briefly, a labeling solution was initially prepared by adding 4 μL of PHK67 to 1 mL of the manufacturer's diluent. A solution of RBCs was prepared by diluting 50 μL of 20% hematocrit RBCs with 950 μL of the manufacturer's diluent. These two solutions were then combined, mixed, and incubated at 37°C for 10 min. This was followed by three washing cycles, comprising centrifugation at 2000 g for 10 min and replacement of the supernatant with an equivalent volume of PBS. Finally, RBCs were resuspended at 30% hematocrit with PBS.

### 2.3 RBC Glycation

RBC glycation was performed with methylglyoxal (MGO) as previously described (Delveaux et al. 2020). RBCs were resuspended at 20% hematocrit in PBS containing 0.1% D‐glucose to mimic physiological conditions. Subsequently, 2 mL of the aforementioned RBC solution was combined and mixed with the MGO solution (Sigma‐Aldrich, Saint‐Quentin, France) at a final concentration of 7.5 mM. All tubes were then incubated at 37°C for 24 h, after which the RBCs were washed three times as previously described and resuspended at 20% hematocrit in PBS.

As a control for glycation, RBCs were treated with a supraphysiological concentration of Glucose (0.8 M) as previously described (Quan et al. 2006). RBCs were resuspended at 40% hematocrit in PBS. Subsequently, 2.6 mL of this solution was mixed with 2.4 mL of 30% Glucose (Lavoisier, Chaix et du Marais laboratory, Paris, France, ATC: B05BA03) to obtain a 20% hematocrit solution, incubated at 37°C for 3 h. After this, RBCs were washed three times as previously described and resuspended at 20% hematocrit.

### 2.4 Shear Stress Gradient Ektacytometry

The deformability of RBCs was investigated using a Laser‐assisted Optical Rotational Deformability Cell Analyzer (LORRCA MaxSis, Mechatronics, Zwaag). To this end, a 20 μL suspension of erythrocytes (20% hematocrit) was mixed with 1 mL of an iso‐osmolar Polyvinylpirrolidone buffer solution (PVP) [viscosity 28.62 mPa/s]. The elongation of RBCs was measured in relation to the intensity of shear stress. Deformability was expressed as the Elongation Index (EI), which was calculated as a function of shear stress intensities ranging from 0.3 to 80 Pa. These measurements provided data concerning RBC elasticity.

### 2.5 Cell Flow Cytometry

Erythrocyte shape, Advanced Glycation End‐products (AGEs) levels, CD47 expression, and eryptosis were measured by flow cytometry using Beckman Coulter CytoFLEX and Cytexpert software (v2.1, Beckman Coulter, Brea, CA, USA).

A specific erythrocyte cell population was selected by gating and could be characterized by its typical location in a forward scatter (FSC) versus a side scatter (SSC) parameter graph.

For AGE level determination, 0.4% hematocrit RBCs were incubated with a rabbit polyclonal AGE antibody (Abcam, ab23722; Cambridge, MA; 1:200 dilution) for 1 h, followed by incubation with anti‐rabbit Alexa 647‐conjugated secondary IgG (Thermo Fisher, REF: A‐21244; Bleiswijk, Netherlands; 1:200 dilution).

For CD47 detection on erythrocytes membrane, samples were stained with CD47 antibodies directed against an APC‐conjugated conformation‐independent antibody (clone B6H12) (Thermo Fisher, Bleiswijk, Netherlands, REF: 17–0479‐42; 1:50 dilution) for 1 h at room temperature.

For eryptosis analysis, Annexin V was used to measure the phosphatidylserine (PS) exposure. In brief, 100 μL of RBCs resuspended at 0.4% hematocrit were incubated with 2 μg/mL of Annexin V‐FITC (Abcam, REF: ab14085) in 100 μL binding buffer for 30 min at room temperature, before flow cytometry analysis. Annexin V fluorescence was measured with a 488 nm excitation wavelength and a 530 nm emission wavelength.

### 2.6 Advanced Glycation End‐Product Dot Blot

Following the initial lysis of RBCs with deionized water, hemoglobin concentration in each sample (CTRL and MGO‐treated) was determined by Drabkin assay by measuring the absorbance at 540 nm with a microplate reader (CLARIOStar Plus, BMG LabTech) using a standard curve of hemoglobin (1–10 μg/μL). For that, each sample was previously diluted at a 1:40 ratio in Drabkin reagent (Sigma Aldrich, REF: D5041) and incubated for 20 min at room temperature.

A volume of 5–10 μL of lysate, corresponding to a total amount ranging from 0.125 to 2 μg of hemoglobin, was loaded onto activated PVDF membrane for each sample (PBS‐incubated and 7.5 mM MGO‐treated RBCs). This was done in conjunction with MGO‐glycated BSA and ribose‐glycated BSA as positive controls. Subsequently, the membrane was blocked for 1 h in PTW buffer (PBS/0.1% Tween 20) containing 5% milk powder prior to an overnight incubation at 4°C with the rabbit polyclonal AGE antibody (Abcam, REF: ab23722; Amsterdam, Netherlands; 1:500) diluted in PTW‐1% milk. The membrane was then washed three times with PTW and incubated with HRP‐Goat Anti‐Rabbit IgG secondary antibody (Jackson ImmunoResearch, REF: 111–035‐144; 1/1000 dilution in PTW‐1% milk) for 4 h at room temperature. After a last step of 3 washing with PTW, revelation was performed using an Amersham ECL Prime kit (Cityva, REF: RPN2232), and chemiluminescence was quantified with Syngene GeneGnome imager (New England Biogroup).

Note the use of two positive controls (annotated “+”, 5 μg of BSA glycated with 7.5 mM ribose (+1) or with 7.5 mM MGO (+2)).

### 2.7 Animals and Ethics

Adult wild‐type (WT) zebrafish (*Danio rerio*, 6 to 18 months old) and from the transgenic line *Tg*(*mpeg1.1:mCherry*) (Ellett et al. 2011) were used. This transgenic line allows visualization of immune cells (microglia/macrophages) in red. They were maintained in standard conditions of light (14 h light/10 h dark), temperature (28°C), pH (7.4) and conductivity (400 μS) in the DéTROI zebrafish facility. All experiments were performed according to French and European Community guidelines for use of animals in Research [86/609/EEC and 2010/63/EU], and approved by the local CYROI animal experimentation ethics committee and the French government (APAFIS: 2024032210495445_v6 and APAFIS: 2018040507397248_v2). Note that in all independent experiments performed in this study, control and treated fish were of the same batch of fish (same age).

### 2.8 Injection of Heterologous (Human) RBCs in the Adult Zebrafish Telencephalon to Mimic the Consequences of ICH (Intra‐Cerebral Hemorrhage)

Adult transgenic zebrafish were anesthetized briefly with 0.02% Tricaine prior to the creation of a small hole in the skull above the medial right telencephalic hemisphere using a sterile needle (BM Microlance 3; 30G^1/2^; 0.3 mm × 13 mm). An intra‐parenchymal injection (2–4 nL) of vehicle (PBS) or 30% hematocrit RBC suspension using a glass microcapillary adapted to a FemtoJet 4 injector (Eppendorf) was made by inserting the microcapillary into the hole. The fish were promptly returned to their aqueous environment, where they rapidly recovered from the anesthetic. They were euthanized at 1, 2, 3, and 4 days post‐injection (dpi) with an overdose of the anesthetic. A particular focus was done at 2 dpi as mpeg1.1‐positive macrophage recruitment was known to peak at 1 day post lesion (dpl) and then decreased at 2 dpl; mpeg1.1 microglia‐positive cells start to be recruited from 1 dpl and peaked at 2 dpl, being the most abundant phagocytic cells at the injury site (Palsamy et al. 2023).

### 2.9 Allogeneic RBCs Injection Into the Adult Zebrafish Telencephalon to Mimic the Consequences of ICH (Intra‐Cerebral Hemorrhage)

Three adult wild‐type (WT) zebrafish were briefly euthanized with 0.02% Tricaine prior to blood harvesting from the ocular cavity using a Hamilton syringe. PBS (1 mL) was placed in a blood collection tube (BD Vacutainer, 4 mL, EDTA K2E), vortexed, and transferred to a new tube. The blood was placed in this EDTA‐containing PBS and incubated with DAPI (1:500) for 10 min. The diluted blood was then centrifuged, washed, and resuspended in 100 μL of PBS (to achieve a target hematocrit of 30%). Since zebrafish RBCs are nucleated, we first verified that the microinjection procedure did not compromise their integrity prior to brain injection. A microinjection droplet was placed onto a microscopy slide, and the integrity of the cell nuclei was confirmed (data not shown).

Three zebrafish were then anesthetized briefly with 0.02% Tricaine prior to intra‐parenchymal injection (2–4 nL) of a 30% hematocrit RBC suspension, as previously described. The fish were promptly returned to their aqueous environment, where they rapidly recovered from the anesthetic. They were euthanized at 2 dpi.

### 2.10 Tissue Preparation, Brain Sampling, Processing and Staining

Following euthanasia, fish were fixed in PBS containing 4% paraformaldehyde (PFA) overnight. The brains were then washed four times for 5 min each in PTW (PBS/0.1% Tween 20) and subsequently embedded in 2% agarose‐PBS. Then, 50 μm thick transverse sections were sliced with a vibratome (VT1000S, Leica) and incubated with DAPI (Sigma‐Aldrich; REF: 10236276001; 1 μg/mL) diluted in PTW. Sections were washed three times and then mounted with Aqua Poly/mount (Polysciences Inc., Warrington, PA, USA). No incubation with anti‐mCherry was required for visualization of the cells.

### 2.11 Stab Wound With Subsequent Hemorrhage to Study Hematoma Clearance Kinetics

Adult WT zebrafish were anesthetized briefly with 0.02% Tricaine prior to telencephalic injury through the right hemisphere using a sterile needle (BM Microlance 3; 30G^1/2^; 0.3 mm × 13 mm). The fish were promptly returned to their tank, where they rapidly recovered from the anesthetic. They were euthanatized after 5 h post‐lesion (hpl) and then at 1, 2, 3, 4, 5, and 6‐days post‐lesion (dpl).

### 2.12 O‐Dianisidine Staining

Following euthanasia, fish were fixed in PBS containing 4% paraformaldehyde (PFA) and stored. The brains were then washed three times for 5 min each in PBS and subsequently embedded in 2% agarose‐PBS. Then, 50 μm thick transverse sections were sliced with a vibratome (VT1000S, Leica), and incubated with O‐dianisidine solution for 5 min (14 mg O‐dianisidine powder dissolved in 10 mL of acetate buffer—10 mM final, pH 4.5—containing 40% EtOH and 0.65% H_2_0_2_). Sections were washed with PBS for 5 min, and then fixed for 10 min in 4% PFA, followed by a 3‐times wash in PTW (PBS/0.1% Tween 20) before being mounted with Aqua Poly/mount (Polysciences Inc., Warrington, PA, USA).

### 2.13 Microscopy

Visualization and imaging were performed with AXIO OBSERVER equipped with Apotome 2 (Zeiss) or laser confocal microscope Eclipse Ti2 (Nikon). Images were then analyzed after similar brightness and contrast adjustments between control and treated groups with Zeiss and/or Image J software.

### 2.14 Cell Counting

Microscopy images were processed with ImageJ/Fiji with the hyperstack module. In order to quantify the number of microglia following the injection procedure, three sections per brain (10X magnification) were analyzed (*n* = 65 brains, divided into 4 groups: 13 brains for vehicle, 33 brains for control RBCs, 13 brains for MGO‐treated RBCs, and 6 brains for Glucose‐treated RBCs), corresponding to a total of 195 sections analyzed. To this aim, microglia were manually counted using the “multipoint” tool for each section. Note that the count of the 3 brain sections for each brain was averaged to perform statistical analyses.

To study phagocytosis, PKH67‐labeled RBC (green) colocalization with microglia/macrophages (red) was analyzed. To this purpose, images of the injection site were made (40X magnification) using the apotome or confocal microscope (2 μm thickness). Similarly, 50 brains from 3 groups were analyzed (33 brains for CTRL RBCs, 13 for MGO‐treated RBCs and 4 for glucose‐treated RBCs). This corresponds to 150 images captured and analyzed (3 per brain). Classification was performed as follows: Merge (yellow) was considered as “phagocytosis,” RBCs in direct contact with microglia were considered as “binding” and RBCs with no contact with microglia were considered as “distant.” Note that the count of the 3 brain sections for each brain was averaged to perform statistical analyses.

Brain hematoma volume was quantified on 3 consecutive sections (50 μm thickness each) from 4 to 5 brains at the respective kinetic time points by measuring the area of blood clot (Volume = area × thickness).

### 2.15 Statistical Analysis

Statistical analyses were performed with GraphPad Prism 8.1.0 using the t‐test and one‐way ANOVA if more than 2 conditions were compared. Results are represented with Standard Errors of the Mean (SEM). A *p*‐value < 0.05 was considered statistically significant.

## 3 Results

### 3.1 Characterization of In Vitro Glycation of Red Blood Cells (RBCs)

Given the small amount of blood available in fish and its rapid coagulation, human erythrocytes were used to study the effect of glycated RBCs on immune cell (microglia/macrophage) recruitment and activity. Glycation of fresh human RBCs was performed using methylglyoxal (MGO), a well‐characterized glycation agent (Delveaux et al. 2020; Tupe et al. 2020).

**FIGURE 1:**
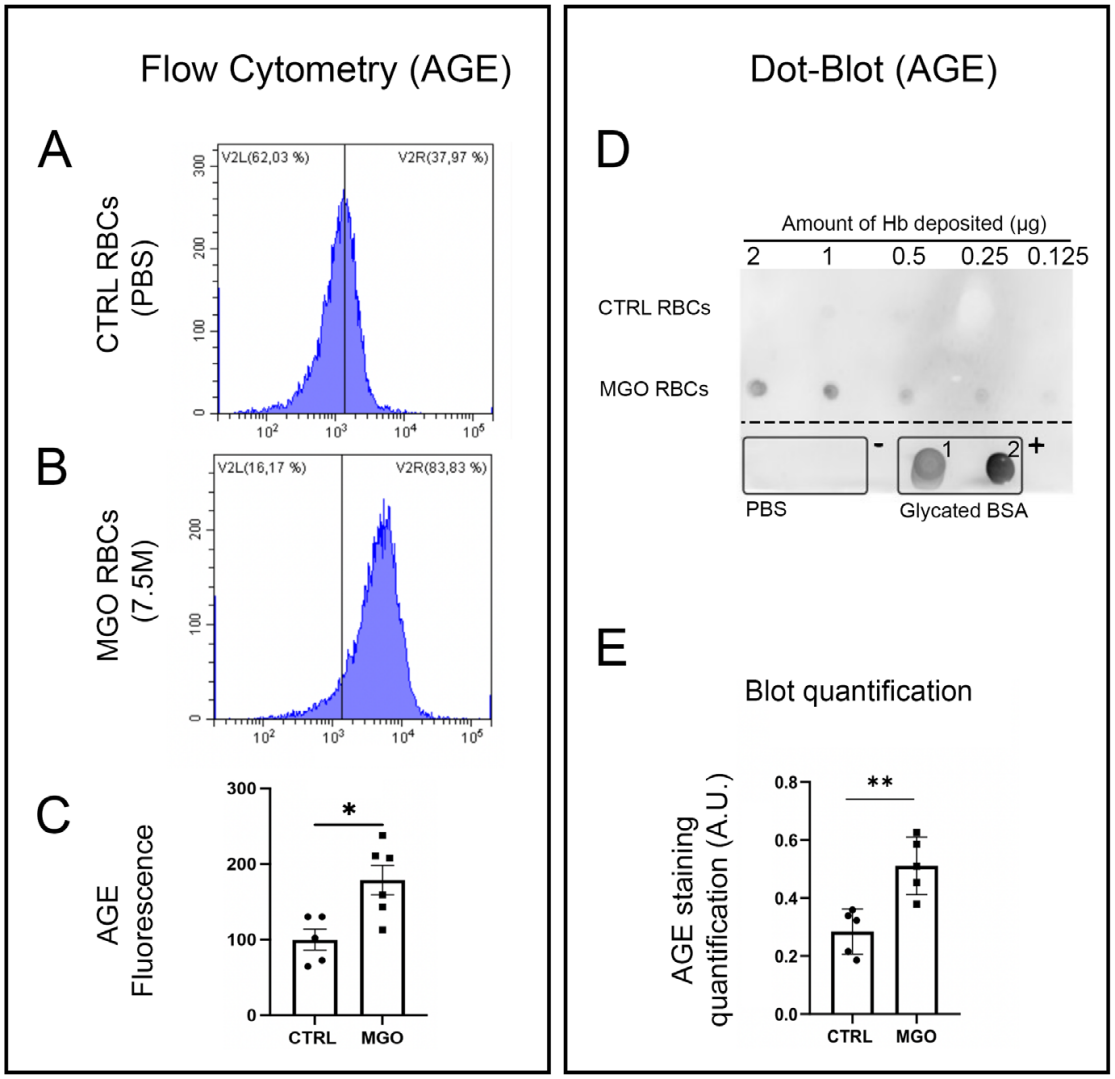
MGO‐treatment induces an increase in AGE content of red blood cells. (A, B) Representative results of AGE detection on erythrocyte membranes by flow cytometry, in PBS‐incubated RBCs (CTRL RBCs) or MGO‐treated RBCs. (C) Quantitative fluorescence analysis of the flow cytometry results showing a significant AGE increase in MGO‐treated condition (*n* = 5–6). (D) Dot‐blot against AGEs in 2 different conditions: PBS‐incubated RBCs (CTRL RBCs) and MGO‐treated RBCs (MGO RBCs) showing an increased AGE signal in glycated condition. Note that a dilution series of the amount of deposited hemoglobin was performed. Below the dashed line, one negative control (annotated “−”, PBS deposit in duplicate) and two positive controls (annotated “+”, 5 μg BSA glycated with 7.5 mM ribose (+1) or with 7.5 mM MGO (+2)). (E) Semi‐quantitative analysis of the dot‐blot showing a significant increase in AGE signal in MGO‐treated RBCs (*n* = 5). Student t‐test (paired, *p < 0.05; **p < 0.01).

After RBC incubation in MGO (7.5 mM) for 24 h, we first verified the glycation of RBCs through the quantification of AGEs by performing cell flow cytometry (Figure 1). As expected, RBCs incubated with MGO (7.5 mM) exhibited increased levels of AGEs (Figure 1A–C). These results were confirmed by dot‐blot, showing a significantly stronger AGE signal in MGO‐treated RBCs compared to PBS‐incubated RBCs (Figure 1D,E). The specificity of these results was established by using negative and positive controls (PBS and glycated BSA, respectively), attesting to the accuracy of our labelling. Consequently, in our experimental conditions, MGO treatment induced glycation of RBCs.

### 3.2 Evaluation of Morphological and Functional Changes in MGO‐Treated Erythrocytes

**FIGURE 2:**
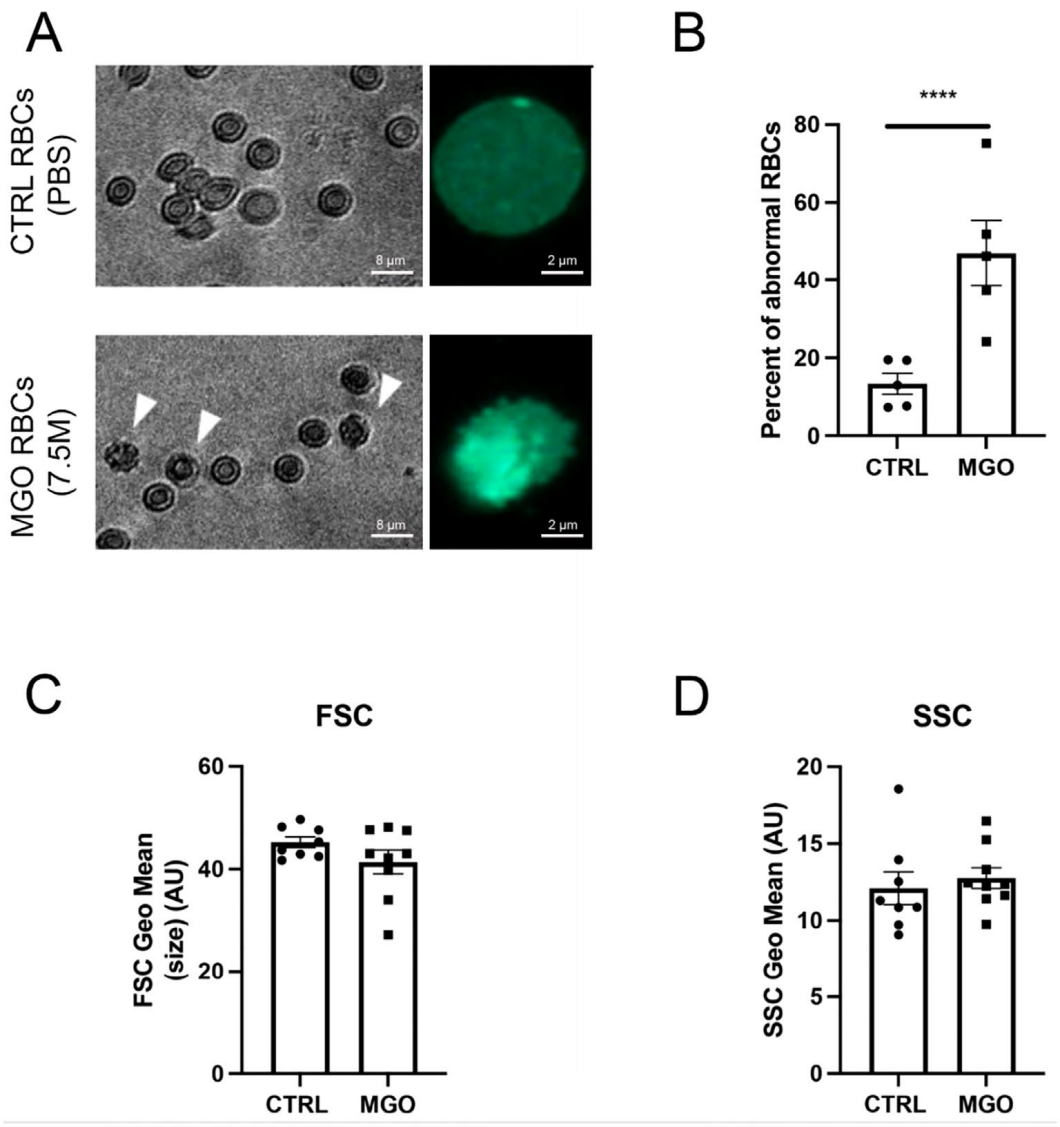
MGO induces morphological changes of red blood cells. Morphological changes including membrane structure alterations (micro‐blebs and spikes), size and granularity were analyzed in PBS‐incubated RBCs (CTRL) and MGO‐treated RBCs (7.5 mM). (A) Representative pictures of the two conditions, with white arrows showing RBC morphological alterations (brightfield and fluorescent RBCs labeled with PKH67). (B) Quantification of abnormal RBC morphology (*n* = 5). (C, D) Cell flow cytometry of CTRL and MGO‐treated RBCs providing morphological characteristics size (FSC) and granularity (SSC) (*n* = 8). Student t‐test (paired, ***p < 0.001). Scale bars: 2 and 8 μm.

We then investigated the impact of MGO‐induced glycation on RBC morphology and deformability. After a 24‐h incubation with MGO (7.5 mM), the morphology of the RBCs was altered with an increased number of structures including micro‐blebs and spikes (Figure 2A, Figure S1). Quantification of these alterations showed that MGO induced a significant increase in RBC shape alterations (Figure 2B). However, flow cytometry analyses did not show any significant impact of glycation on RBC size (FSC) and granularity (SSC) (Figure 2C,D), although they looked microscopically smaller. These morphological alterations are characteristics of senescent/glycated RBCs (Babu and Singh 2004; Mortas et al. 2021; Loyola‐Leyva et al. 2022). We also noted that the supernatant obtained by the first centrifugation of RBCs after MGO incubation displayed consistently signs of hemolysis probably due to increased fragility following the glycation process (Figure S2). Similar results were obtained with our positive control using supraphysiological concentration of glucose (Figure S2).

**FIGURE 3:**
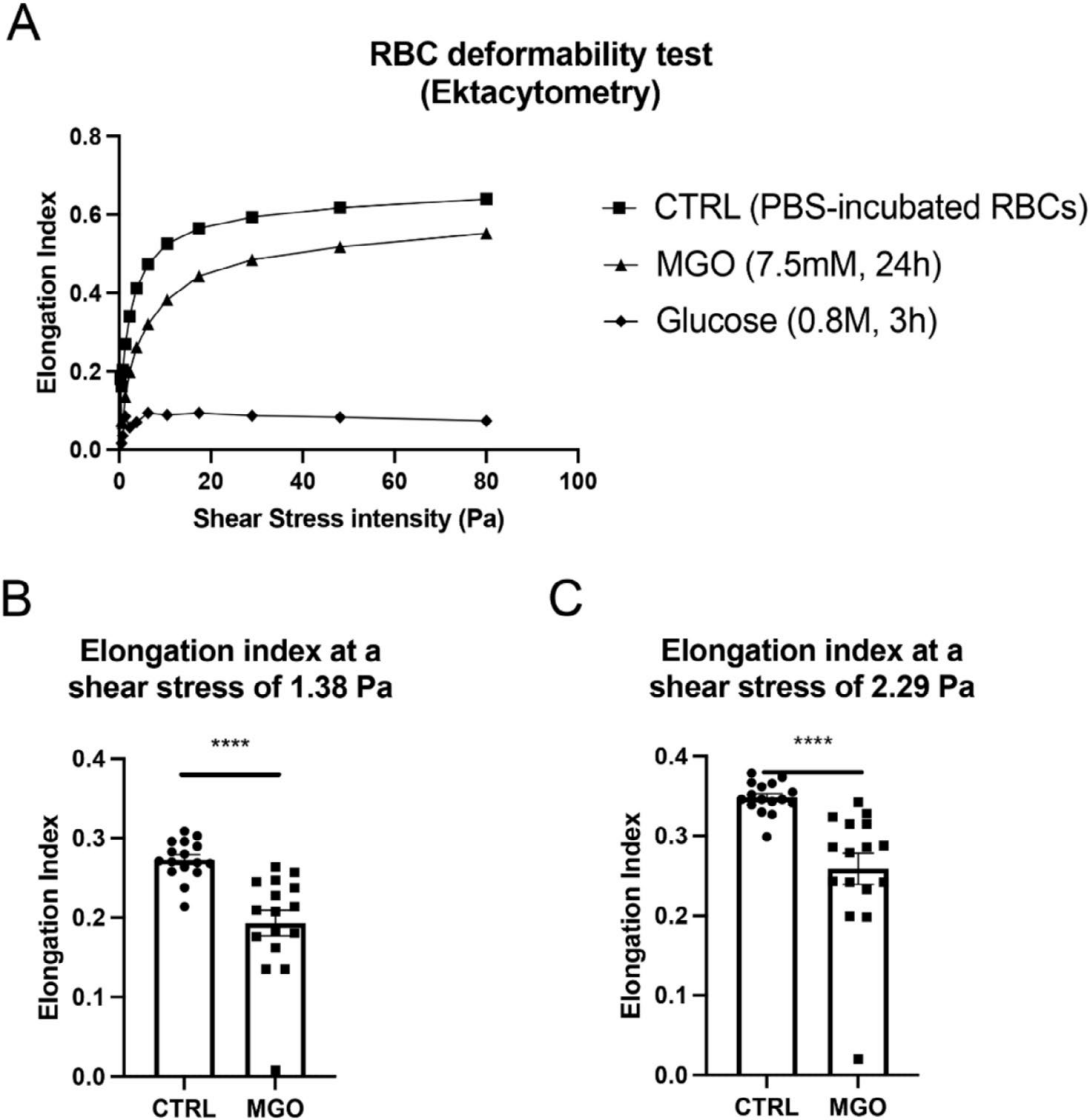
Glycated RBCs display impaired deformability. (A) Representative curves showing deformation as Elongation Index (EI) as a function of increasing shear stress intensities, measured by ektacytometry. MGO‐Glycated RBCs (triangles) showed a decrease in their deformability capacity compared to controls (squares). These results were confirmed by a positive control (RBCs treated with 0.8 M glucose for 3 h, diamonds). (B, C) At shear stress usually found in cerebral vessels around 2 Pa (1.38–2.29 Pa), the elongation index was significantly decreased in Glycated RBCs (*n* = 16). Statistical analysis: Student paired *t*‐test (***p < 0.001).

We next studied the rheological properties of RBCs by examining their deformability using ektacytometry. As shown in Figure 3A, RBCs incubated with 7.5 mM MGO exhibited a lower deformability capability than their respective controls (PBS‐incubated RBCs, 24 h). As it was previously shown that incubation with high concentrations of glucose (0.137–0.8 M) led to strong alteration of RBC membrane (phosphatidylserine exposure and deformability) (Quan et al. 2006; Turpin et al. 2020), we assumed that such conditions could be used as a positive control. Indeed, we observed that RBCs treated with 0.8 M glucose for 3 h induced a strong decrease in erythrocyte deformability compared to native RBCs (PBS‐incubated for 3 h, not shown as it perfectly overlaps with PBS‐ RBCs incubated for 24 h) and is also associated with increased hemolysis (Figure 3A and Figure S2). Such alterations are representative of the diabetic conditions (Babu and Singh 2004; Keymel et al. 2011; Ebenuwa et al. 2024). Interestingly, at around 2 Pa (1.38–2.29 Pa) corresponding to the level of physiological shear stress that can be observed in the middle cerebral artery (Zhao et al. 2015; Gao et al. 2021), glycated erythrocytes have a significantly impaired deformability (Figure 3B,C).

### 3.3 Identification of Pro‐Eryptosis and “Eat Me” Signals in Glycated RBCs

**FIGURE 4:**
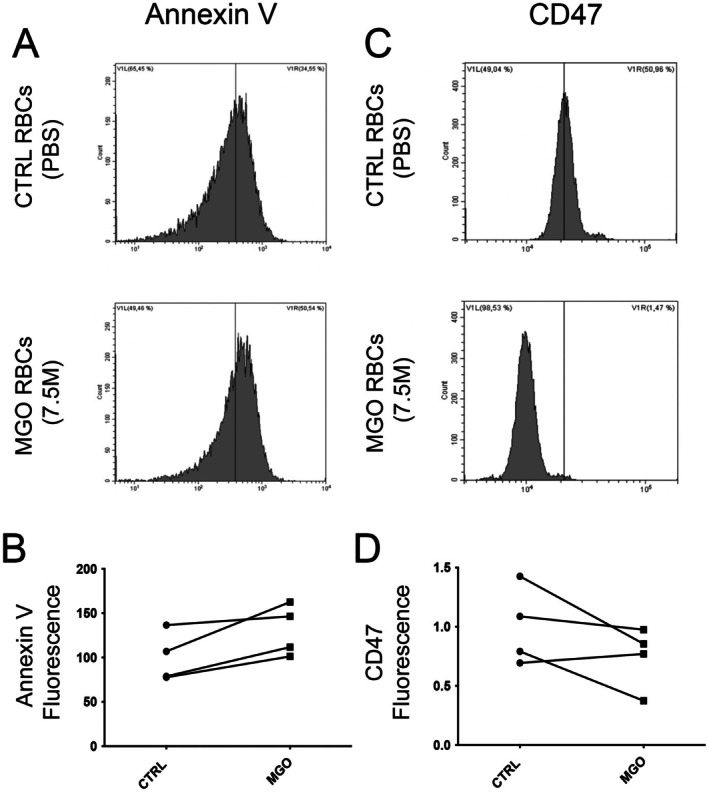
Glycation tends to increase eryptosis and senescence of red blood cells. (A, B) Representative flow cytometry histograms and quantification of annexin V‐FITC fluorescence intensity measurement in CTRL and MGO‐treated conditions indicating a slight increase in phosphatidylserine exposure, an eryptosis marker. (C, D) Representative flow cytometry histograms and respective quantification of CD47 fluorescence intensity measurement in CTRL and MGO‐treated conditions indicating a slight decrease in glycated condition. Note that CD47 signal represents a “don't eat me signal” used as a senescence marker. Note that no significant difference was observed between conditions (Paired *t*‐test, *p* = 0.059 for Annexin V and 0.53 for CD47).

Finally, as glycation can induce apoptosis, phosphatidylserine (PS) flip‐flop as a main sign of eryptosis (RBC apoptosis) (Boulet et al. 2018; Waggiallah 2020) was investigated using annexin V‐FITC. Although no significant difference was observed between native and glycated RBCs, a slight increase in annexin V fluorescence was detected after MGO treatment (Figure 4A,B), suggesting an increased PS exposure (“eat‐me signal”). In contrast, membrane CD47, a “don't eat‐me signal” (Fang et al. 2022), slightly decreased in MGO‐treated RBCs compared to its respective control (Figure 4C,D). This reduction reflects RBC senescence, likely to favor phagocytosis. Although these results did not reach significance, they indicate a tendency to an increase in eryptosis/senescence during the glycation process.

Taken together, these data demonstrate that in our experimental conditions, MGO treatment results in (i) RBC glycation, (ii) morphological changes, and (iii) a decreased deformability capacity associated with eryptosis/senescence features. These results are consistent with impairments known in diabetic conditions (Babu and Singh 2004; Mortas et al. 2021; Ebenuwa et al. 2024).

## 4 Development of an Innovative *In Vivo* Model of Intracerebral Hemorrhage (ICH)

In a next step, we investigated the impact of RBC glycation on the recruitment of microglia/macrophages using zebrafish as a relevant model of brain repair (März et al. 2011; Kizil et al. 2012; Schmidt et al. 2014; Diotel et al. 2020; Ghaddar, Lübke, et al. 2021). To this aim, we set up a new model to mimic the consequences of ICH by directly injecting RBCs into the telencephalon of adult zebrafish and investigated immune cell trafficking and erythrophagocytosis.

**FIGURE 5:**
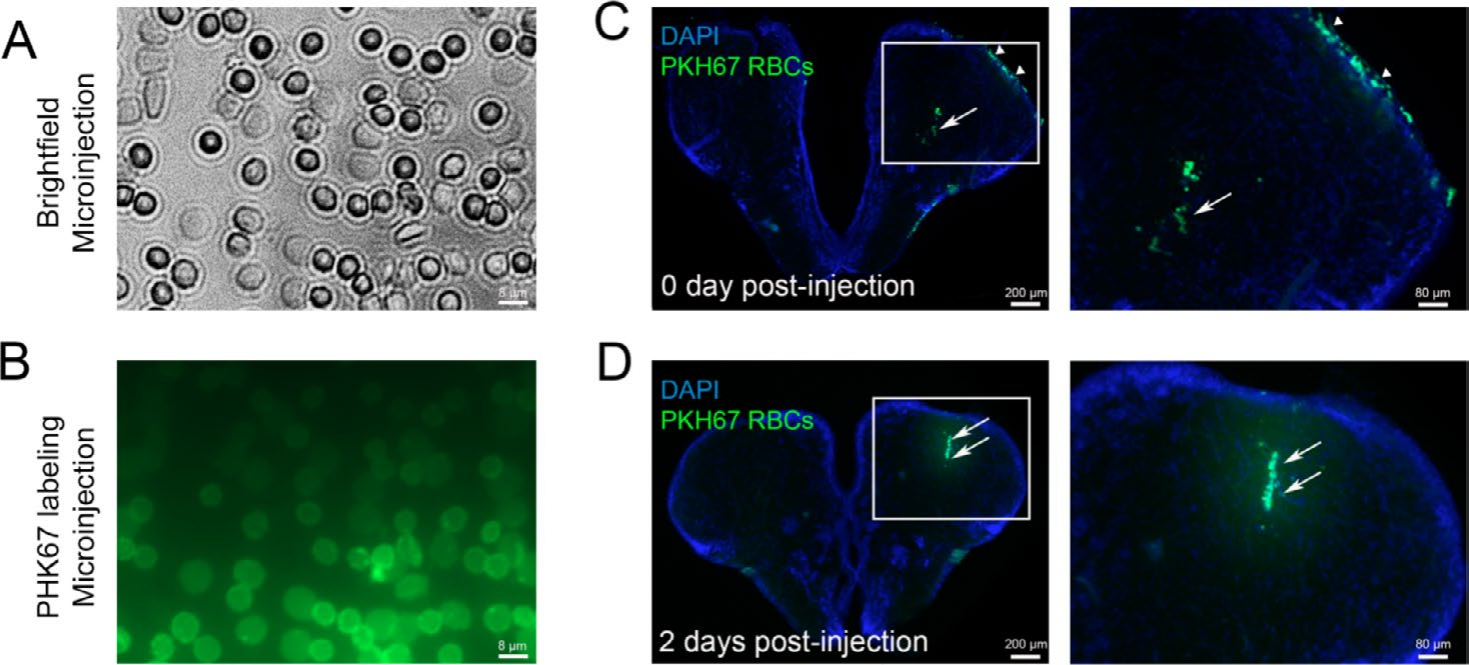
Efficient injection and persistence of PKH67‐labeled human RBCs in the telencephalon of adult zebrafish. (A, B) General brightfield view (A) and fluorescence (B) of PHK67‐labeled RBCs after passing through the microcapillary injection system. Note that the integrity of RBCs is well preserved and that they are efficiently labeled. (C, D) Visualization of labeled RBCs immediately after the intra‐telencephalic injection (C) and 2 dpi (D). The blue color corresponds to DAPI nuclei counterstaining. Arrows point to RBCs at the injection site, while arrowheads indicate RBC spillage. Scale bar: 80 and 200 μm. The experiment was repeated at least 3 times with similar results.

After RBC labeling with PKH67 (Figure 5A,B), fish were anesthetized and a small hole was made in the skull just above the right telencephalic hemisphere. Then, a glass microcapillary filled with freshly labeled RBCs was introduced within the telencephalic parenchyma and erythrocyte suspension was injected. As shown in Figure 5C,D, PKH67‐labeled RBCs were observed in the brain parenchyma immediately after injection and persisted at least until 2 days post‐injection (dpi, arrows), demonstrating the efficacy of the injection. In some cases, a ventricular spillage has been observed (Figure 5C, arrowheads). Interestingly, the persistence of RBCs at 2 dpi allows us to investigate the recruitment of immune cells (microglia/macrophages) at the injected site (Figure 5).

**FIGURE 6:**
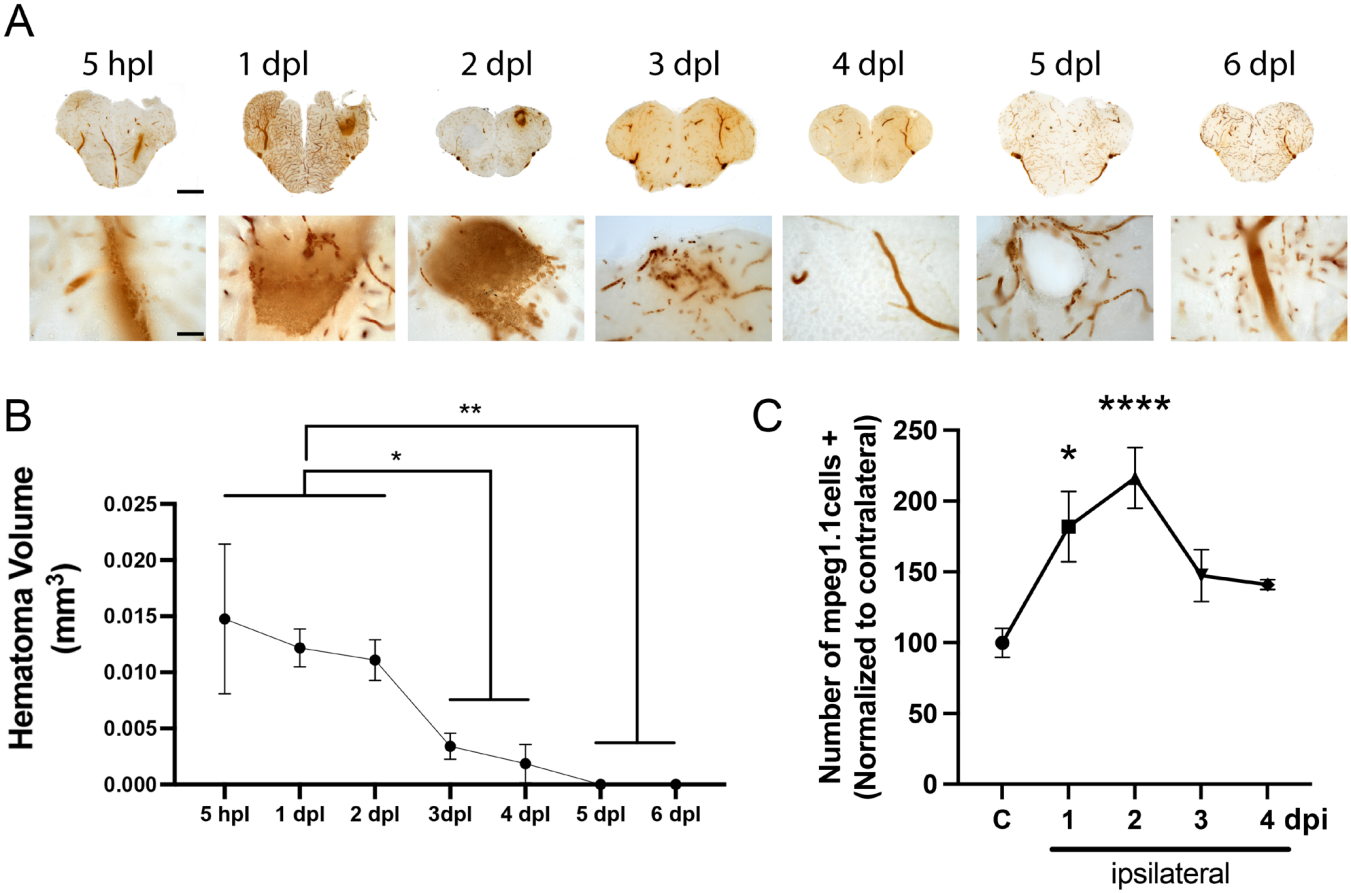
Hematoma clearance and recruitment of immune cells after human RBC injection. (A) Kinetics of hematoma resorption in the telencephalic parenchyma following a stab wound injury using a needle (from 5 h post‐lesion ‐hpl‐ to 6 days post‐lesion ‐dpl‐) by O‐dianisidine staining. Hematoma is obvious at 5 hpl, 1 and 2 dpl. (B) Quantification of the hematoma volume following brain injury demonstrating a significant decrease from 3 dpl compared to the 2 first days. One‐Way Anova (*n* = 3–15). (C) Quantification of mpeg1.1‐positive cells from 1 to 4 days post‐injection (dpi) of human RBC in the telencephalon. Data were normalized according to the number of mpeg1.1 cells in the contralateral hemispheres. Immune cells were significantly increased at 1 and 2 dpi. One‐Way Anova (*n* = 3–15). *p < 0.05; ***p < 0.01; ****p < 0.0001. Scale bar: 270 μm for low‐magnification images and 20 μm for high‐magnification images.

Two days post‐injection/lesion represents a particularly relevant time point in zebrafish, as it corresponds to the early phase of immune cell recruitment/activation, involving microglia and macrophages, following various types of telencephalic injury (Kyritsis et al. 2012; Palsamy et al. 2023; Fernezelian et al. 2025). As well, in such traumatic injuries, blood hematoma appears to be cleared within the first days. In order to better define the time windows of analysis, we first decided to analyze the clearance of brain hematoma in an extensive model of hemorrhage in zebrafish. To this aim, we performed O‐dianisidine staining to label red blood cells at different time points after a telencephalic stab wound injury induced by inserting a 30G needle through the skull. As shown in Figure 6, the brain hematoma is clearly visible from 5 h post‐injury to 2 dpl, before being progressively cleared from 3 dpl (Figure 6A). Quantification of the hematoma volume confirmed RBC clearance in 3 days (Figure 6B). This timing perfectly fits with the recruitment of microglia/macrophage (März et al. 2011; Palsamy et al. 2023; Fernezelian et al. 2025; Kyritsis et al. 2012). However, we questioned whether the kinetics of microglia/macrophage recruitment induced by stab wound injury overlapped with those triggered by our RBC injection paradigm. To this aim, we took benefit of the *Tg(mpeg1.1:mCherry)* zebrafish to visualize microglia and macrophages. Following the injection of human RBCs, the recruitment of mpeg1.1‐positive cells was analyzed, revealing a significant increase at 1 dpi, peaking at 2 dpi before reaching basal levels. Therefore, 2 dpi represents a pivotal stage for immune cell recruitment and phagocytic activity analysis following RBC injection in zebrafish.

## 5 Impact of RBC Presence Within the Brain Parenchyma on mpeg1.1‐Positive Cell Recruitment

**FIGURE 7:**
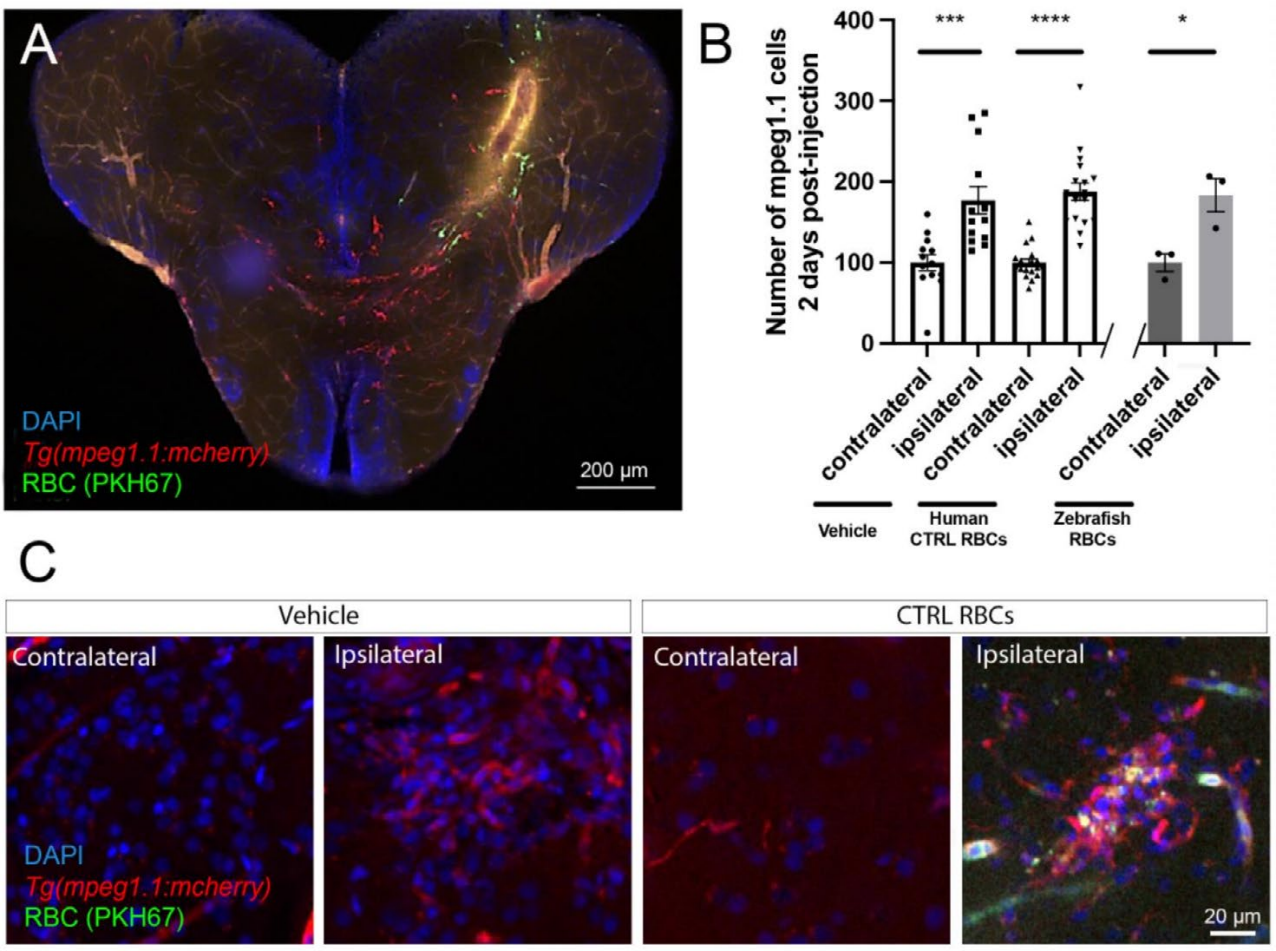
Human and zebrafish RBC injection in the telencephalon does not impact the recruitment of mpeg1.1‐positive cells at the injection site. (A) Representative picture of human RBC injection (green) in the telencephalon of *Tg(mpeg1.1:mCherry)* fish at 2 dpi. (B) Percentage of mpeg1.1‐positive cells in both contralateral and ipsilateral hemispheres of vehicle, human RBC, zebrafish RBC‐injected animals at 2 dpi. Both conditions resulted in a significant increase in mpeg1.1‐positive cells without difference between their recruitment. Scale bar 200 μm. *n* = 3–18 brains/condition. (C) Representative high magnification pictures of vehicle and CTRL human RBC‐injected animals showing increased number of mpeg1.1‐positive cells (red) in ipsilateral hemispheres compared to contralateral ones. One‐way ANOVA and Student *t*‐test (*p < 0.05; ***p < 0.001; ****p < 0.0001).

After ensuring the efficacy of the method for injecting RBCs within the brain parenchyma and ascertaining the time of analysis following injection, we evaluated the impact of human RBC presence on immune cell recruitment compared to a control injection. To this aim, we used *Tg(mpeg1.1:mCherry)* zebrafish to visualize microglia and macrophages. In both groups injected with the vehicle or RBCs, a significant increase in mpeg1.1‐positive cells was observed in the ipsilateral hemisphere (Figure 7B). However, no significant difference was observed in mpeg1.1‐positive cell recruitment following vehicle or RBC injection. Firstly, this result demonstrates an increased immune recruitment after microlesion/injection, regardless of the substance injected. Secondly, this demonstrates that the presence of heterologous human RBCs did not significantly impact the recruitment of microglia/macrophage in regard to the lesion induced by the insertion of the needle. Importantly, although we cannot ascertain that the presence of human RBCs in the telencephalon has no impact on the immune system, we showed that the injection of zebrafish RBCs triggers a similar recruitment of mpeg1.1‐positive cells (Figure 7B). Together, these data allow us to further investigate the impact of human RBC glycation on immune cell recruitment in our model.

## 6 Glycation of Red Blood Cells Favors Phagocytosis Without Impacting the Number of mpeg1.1‐Positive Cells at the Injection Site

**FIGURE 8:**
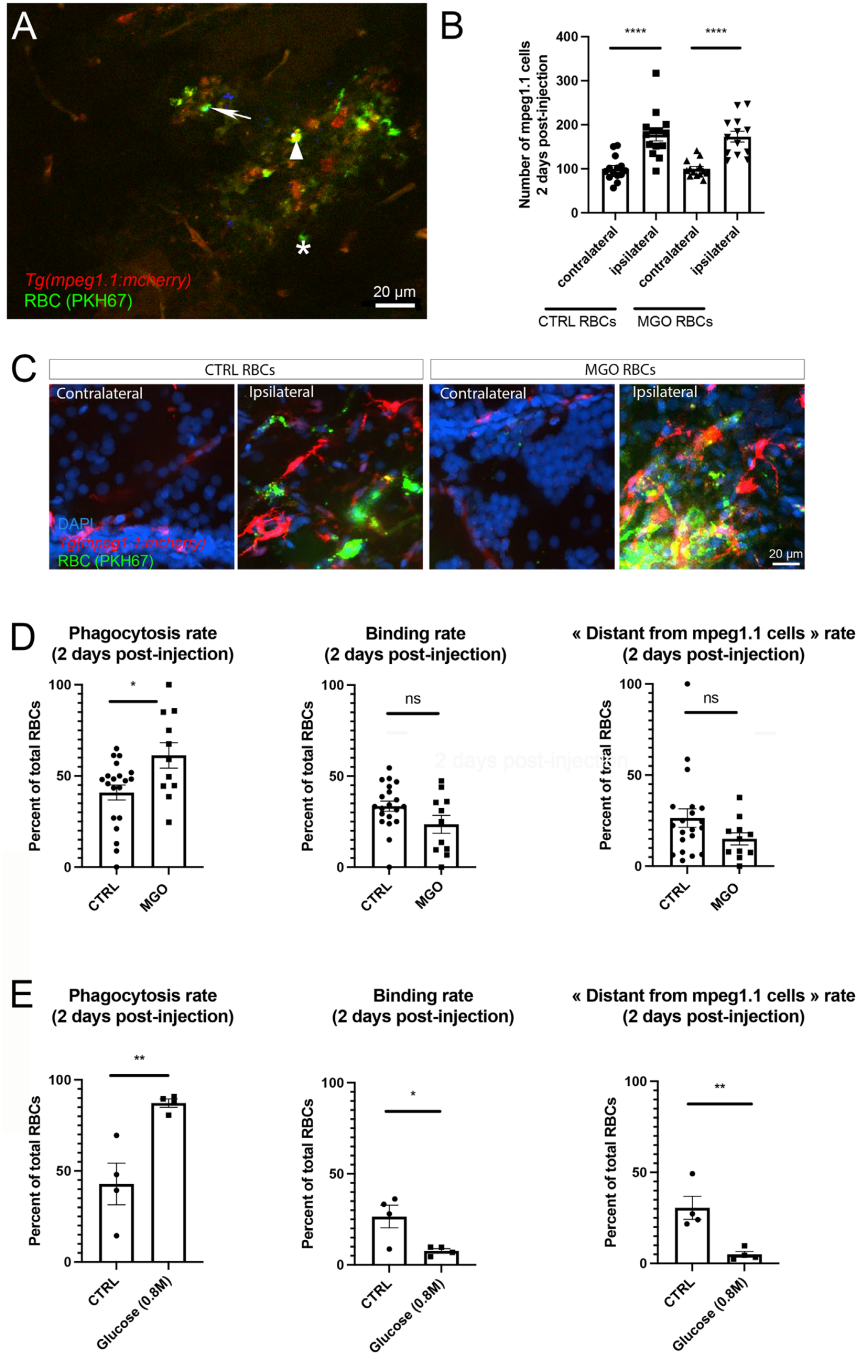
Glycation of RBC enhances erythrophagocytosis without affecting microglial/macrophage recruitment. (A) Representative confocal picture (thickness 2 μm) in the injected hemisphere showing RBC position (green) relative to mpeg1.1‐positive cells (red) around the injection site at 2 dpi. Note that merge (yellow, arrowhead) was considered as “phagocytosis”; “nearby” (arrow) represents “binding” of RBCs to mpeg1.1‐positive cells; and “distant” (asterisks) the non‐bound RBCs. (B) Number of mpeg1.1‐positive cells in the contralateral and ipsilateral hemispheres following CTRL and MGO‐treated RBC injection. (C) Representative high magnification pictures of contralateral and ipsilateral telencephalon, after injection of CTRL and MGO RBCs. (D, E) Percentage of phagocytosis, binding rate and distant RBCs in MGO‐ (D) and glucose‐treated (E) conditions, relative to their respective controls. Scale bar: 20 μm. *n* = 11–20 for MGO experiments and *n* = 4 for glucose experiments. One‐way ANOVA and Student paired t‐test (*p < 0.05; ***p < 0.001; ****p < 0.0001).

We next investigated the consequences of RBC glycation on microgliosis. To this end, PBS‐incubated (CTRL) and MGO‐treated RBCs labeled with PKH67 were injected in the telencephalon of *Tg(mpeg1.1:mCherry)*, and immune cell recruitment and phagocytosis were analyzed. As shown in Figure 8, the recruitment of mpeg1.1‐positive cells was increased in the injected hemispheres in both conditions, without significant difference (Figure 8A,B). These data support the fact that glycated RBCs did not impact immune cell recruitment.

We next investigated erythrophagocytosis in the close vicinity of the injection site. To this aim, we established a score that identifies the RBC position in relation with mpeg1.1‐positive cells. Through confocal microscopic analyses, the colocalization of labeled RBCs (green) and mpeg1.1 cells (red) resulting in a yellow color was considered as phagocytosis (Figure 8A). RBCs surrounding mpeg1.1‐positive cells were considered as “binding” and the other RBCs far from mpeg1.1. cells were classified as “distant” (Figure 8A, see arrow, arrowhead and asterisk). The quantification of these parameters demonstrated that the majority of MGO‐glycated RBCs were phagocytosed (up to 60%), and that their phagocytosis was significantly higher than for control RBCs (Figure 8D). Also, the proportion of “distant” RBCs tended to be lower with glycation. These results suggest that glycation favors microglial/macrophage recruitment and hence glycated RBC phagocytosis.

We then sought to confirm these results using another glycation method based on erythrocyte incubation with supraphysiological concentrations of glucose. In the literature, RBC glycation and eryptosis can be quickly and efficiently achieved by incubation in 0.8 M of glucose for 3 h at 37°C (Quan et al. 2006). In our experimental conditions, despite the stronger effect of glucose‐mediated glycation, this treatment resulted in RBC alterations similar to MGO (PS flip‐flop, CD47 decrease, AGE increase, and morphological abnormalities) (Figure S3). The injection of such glucose‐treated RBCs reinforced the results obtained by MGO. Indeed, glucose‐RBCs were more prone to be phagocytosed relative to MGO‐RBCs (~87% vs. ~61%), resulting in a significantly reduced number in RBC in “binding” and “distant” positions (Figure 8E; ~8% vs. 23% for binding and ~5% vs. ~15% for distant).

In conclusion, we set up a method based on RBC injection in the telencephalon to mimic the consequences of a hemorrhagic stroke in adult zebrafish. We demonstrate that the presence of intraparenchymal RBCs results in the active recruitment of immune cells within the first two days and leads to erythrophagocytosis. In these experimental conditions, glycation of RBCs either by MGO or by high glucose concentrations increases their propensity to be phagocytosed by microglial cells and macrophages. This result highlights that RBC glycation disrupts phagocytic mpeg1.1 cell behavior and RBC clearance, raising the question of such impairments on the consequences of hemorrhagic strokes.

## 7 Discussion

In this work, we developed a model of MGO‐induced glycation of human erythrocytes and we demonstrated that glycated RBCs exhibited properties commonly found in diabetes such as higher levels of glycation, impaired deformability, morphology, and increased eryptosis marker (Tupe et al. 2020; Mortas et al. 2021; Loyola‐Leyva et al. 2022; Ebenuwa et al. 2024). We also established a zebrafish model of the consequences of an ICH to investigate the impact of glycation on brain repair involving microglia/macrophage recruitment and associated phagocytosis. Although RBC did not affect the recruitment of these immune cells in the injected hemisphere compared to vehicle injection, significant enhanced phagocytosis was observed when glycated RBCs were injected relative to native non‐glycated RBCs. These results were reproduced by using high glucose concentrations as another well‐established glycating agent. Thus, our study shows that RBC glycation promotes enhanced phagocytosis that may influence brain regeneration.

### 7.1 In Vitro Glycation of RBCs Allows to Mimic the Modifications Found in Diabetes

For our experiments, we chose methylglyoxal (MGO), a potent glycating agent that can be produced during the glycolysis process. This compound is reported to be increased in plasma of diabetic patients and is frequently associated with microvascular cerebral disorders (Schalkwijk and Stehouwer 2020; Tupe et al. 2020; Matafome et al. 2017). It is also suspected to have a deleterious impact on auto‐immune and neurodegenerative diseases such as multiple sclerosis (Wetzels et al. 2019). The short incubation time of RBCs with MGO (24 h) allowed us to avoid the confounding aging process that occurs in longer glycation protocols, without drastically impacting the viability and functionality of RBCs. We demonstrated that MGO treatment (7.5 mM, 24 h) efficiently induced the glycation of RBCs, as evidenced by the presence of AGEs in dot blot and flow cytometry analyses. These results are in accordance with previous studies (Delveaux et al. 2020; Tupe et al. 2020).

We also showed that MGO incubation led to specific morphological changes in glycated RBC shape such as microbleb‐like and/or echinocyte‐like structures (Feng 2016; Mortas et al. 2021). Microblebs in diabetic conditions have been described using electronic microscopy in several studies (Buys et al. 2013; Feng 2016; Loyola‐Leyva et al. 2022). The percentage of echinocytes (a characteristic feature of RBCs) has also been reported to be increased in diabetic blood samples (Mortas et al. 2021). Taken together, we demonstrated that our MGO treatment disrupts the physiological morphology of RBCs similarly to diabetic conditions. Surprisingly, although microscopic analysis showed a trend towards smaller size of MGO‐treated erythrocytes, this was not confirmed by cell cytometry.

In addition, MGO‐glycated erythrocytes display lower deformability capacities. This is particularly evident at physiologic shear stress levels (around 2 Pa), which can be encountered in cerebral vessels (ex: middle cerebral artery territory, where ICH often occurs) (Zhao et al. 2015; Garcia‐Polite et al. 2017; Gao et al. 2021). These data were also corroborated by glucose‐mediated glycation. A reduced deformability capacity of RBCs was also described during the glycation process in different studies (Babu and Singh 2004; Shin et al. 2007; Delveaux et al. 2020; Ebenuwa et al. 2024). Changes in the erythrocyte membrane could explain their increased fragility during the glycation process and the resulting hemolysis, as shown in Figure S2.

Interestingly, in our experimental conditions, MGO treatment tends to increase eryptosis, a specific form of RBC apoptosis characterized by phosphatidylserine (PS) flip‐flop allowing their labelling by annexin V (Fang et al. 2022). Eryptosis is known to promote RBC phagocytosis, notably after ICH (Liu et al. 2022). This process can take place to protect the brain parenchyma from the release of toxic blood components that would be deleterious if not removed. Eryptosis is initiated by various steps including increased “eat‐me signals” (i.e., PS flip‐flop), and decreased levels of “don't eat‐me” signal (i.e., CD47). Here, we identified a slight decrease in CD47 expression after MGO treatment and higher PS exposure. This supports the idea that glycation can promote erythrophagocytosis, which was further confirmed in vivo following control versus glycated RBC injection in our study. Finally, all results obtained by MGO‐mediated glycation were confirmed by glucose treatment, ascertaining the robustness of our innovative glycation method.

In conclusion, our MGO‐mediated model of RBC glycation appears efficient and relevant to the changes seen in diabetic conditions (Turpin et al. 2020), while avoiding the additional senescence associated with a longer procedure. Furthermore, although MGO glycation induces relatively moderate effects on RBC features, it nevertheless promotes their active phagocytosis as shown after their parenchymal injection.

### 7.2 Modeling the Consequences of ICH on Initial Phase of Brain Repair Mechanisms Using Zebrafish: Focus on mpeg1.1 Cell Recruitment

In order to study the consequences of ICH and RBC glycation, we take advantage of zebrafish given its remarkable capacity for cerebral regeneration and its suitability for investigating metabolic diseases in the brain (März et al. 2011; Ghaddar and Diotel 2022). Zebrafish share 70%–85% of genes with humans and display well‐conserved brain regions, functions and regenerative mechanisms (Howe et al. 2013; März et al. 2011; Kizil et al. 2012; Chen et al. 2021; Ghaddar, Lübke, et al. 2021). We consequently sought to develop an efficient method to inject RBCs within zebrafish brain parenchyma to mimic the consequences of ICH, considering the actual absence of a simple and efficient zebrafish model to study neuro‐restoration after a hemorrhagic injury in adult fish (Alharbi et al. 2016).

During brain hemorrhage, total blood (i.e., erythrocytes, peripheral blood mononuclear cells, plasma proteins) is spread within the parenchyma. By contrast, our model allows focusing research on native or glycated erythrocyte impact in ICH without interference from other blood components. Indeed, erythrocytes are hypothesized to be a main source of secondary brain injury by inducing, among others, toxicity, oxidative stress, and inflammation occurring during hemolysis. Their clearance is a key process in improving neurological outcomes. Herein, we confirmed the strong capability of zebrafish for brain repairment following large lesion by demonstrating efficient clearance of hematoma following the conventional stab wound injury model. Such a process is correlated to the recruitment of phagocytic cells (microglia and macrophages) as previously shown in the literature (März et al. 2011; Palsamy et al. 2023; Kyritsis et al. 2012; Fernezelian et al. 2025).

To study phagocytosis, we used the transgenic zebrafish line *Tg(mpeg1.1:mCherry)* which allows visualization of microglia/macrophages in red. After telencephalic injury, it is well established that a first wave of infiltration of peripheral macrophages (mpeg1.1‐positive and 4C4‐negative) occurs, peaking at 1dpl and decreasing massively at 2 dpl. In parallel, microglia (mpeg1.1‐positive and 4C4‐positive) are initially recruited within the first hours after injury, increasing abundantly from 1 dpl and peaking at 2–3 dpl (Palsamy et al. 2023; März et al. 2011). In our microlesioning/RBC injection model, we also confirmed a significant increase in mpeg1.1‐positive cells at 1 and 2 dpi suggesting that most mpeg1.1‐positive cells may correspond to microglia at 2 dpi (Palsamy et al. 2023; März et al. 2011). However, it would be important to specify the exact nature of these cells in this particular context of ICH.

In our model, we observed an increase of mpeg1.1‐positive cell recruitment in the injected hemisphere compared to the contralateral one, irrespective of the injected material (vehicle or control RBCs). In addition, when zebrafish (allogeneic) RBCs were microinjected into the telencephalon, the recruitment pattern of mpeg1.1‐positive cells was comparable to that observed following vehicle or human RBC injections. These results clearly suggest that the recruitment of phagocytic cells is due to the microinjection process rather than the injection of heterologous RBCs.

### 7.3 Glycation of RBCs Enhances Erythrophagocytosis Without Affecting the Number of Phagocytic Cells Recruited

To study the impact of RBC glycation on the recruitment of mpeg1.1‐positive cells at 2 dpi, we performed intraparenchymal injection of PBS‐incubated or MGO‐treated RBCs in the telencephalon. A comparable increase in the number of mpeg1.1‐positive cells was observed in the injected hemispheres, irrespective of the glycation status of RBCs.

However, a higher rate of phagocytosis was observed with glycated RBCs compared to native ones (~61% vs. ~41%) at 2 dpi. Independent of the glycation status, these data also highlight the capacity of the zebrafish brain to actively clear RBCs from the cerebral parenchyma as 41% of RBCs are already involved in a phagocytic process at solely 2 dpi. These data fit well with the absence of detectable RBCs that could persist after telencephalic stab wound injury of the telencephalon from 3 to 5 days post‐lesion (data not shown).

In agreement, a decrease in “binding” and “distant” glycated RBCs was observed compared to control RBCs. This finding indicates that mpeg1.1‐positive cells exhibit increased or accelerated phagocytic activity for glycated versus control RBCs. Under MGO glycation, this higher phagocytic rate is correlated with the increased PS exposure and decreased levels of CD47 (“don't eat me” signal), although not statistically significant. We can consequently consider that glycation promotes eryptosis that subsequently favors erythrocyte clearance by phagocytic cells. Similarly, we independently verified these data with another series of experiments using glucose as a glycating agent (0.8 M for 3 h at 37°C). This protocol appears more drastic as it induces more severe defects than MGO (ex: morphology, deformability, markers promoting eryptosis, hemolysis), and subsequently results in stronger phagocytosis than with MGO‐mediated glycation (~87% for glucose vs. ~61% for MGO). We can also notice that our methodology is reproducible as the phagocytic rate of control RBCs remains very similar between these two independent experiments (~43% for glucose vs. ~41% for MGO).

In other pathological processes, glycation of RBCs is known to increase phagocytosis by different cell types including macrophages (physiological clearance), endothelial cells, and vascular smooth muscular cells during atherosclerosis (Catan et al. 2019). It is notably shown that MGO impairs macrophage efferocytosis in diabetic wounds (Zhu et al. 2025).

Consequently, these results highlight the fact that RBC glycation (MGO‐ and glucose‐mediated) has no impact on the increased number of mpeg1.1‐positive cells recruited at the injected site, but favors erythrophagocytosis. Microglia recruitment and activation at 2 days post‐injury is consistent with the literature in zebrafish and mammals (rodent/human) (Ghaddar, Lübke, et al. 2021; Zhang et al. 2021), as well as following ICH in rodent, human, and zebrafish larva (Guo et al. 2022; Liu et al. 2022; Puy et al. 2022; Crilly et al. 2018).

Considering the deleterious impact of diabetes/hyperglycemia during ICH, the increase in erythrophagocytosis was quite unexpected as it suggested a better clearance of toxic elements. However, it is conceivable that glycation‐accelerated erythrophagocytosis could overwhelm the capacity of microglia and macrophages, leading to the accumulation of large amounts of RBC degradation products, particularly iron, which might in turn contribute to cellular stress and potentially favor apoptosis induced by iron (ferroptotic pathways) (Liu et al. 2025). This may finally increase cerebral inflammation and oxidative stress, resulting in stronger brain damage. Also, these data raise the question of the possibility of erythrophagocytosis by other cell types such as neurons, oligodendrocytes, and astrocytes and the impact of efferocytosis/iron toxicity in such cells.

### 7.4 Limitations of the Study

In this study, we developed a zebrafish telencephalon injection model using RBCs to investigate the impact of glycation on phagocytic responses. Unexpectedly, all injections such as vehicle, human RBCs (glycated or not), and zebrafish RBCs have resulted in comparable recruitment of mpeg1.1‐positive cells. It is important to note that the number of RBCs injected in this model is relatively low compared to mammalian models of autologous blood injection (MacLellan et al. 2008; Puy et al. 2025). Therefore, it is plausible that such a limited amount of RBCs in the parenchyma does not exceed the inflammatory response triggered by the microinjection procedure itself. Furthermore, in our experimental design, we used isolated RBCs devoid of other blood components (e.g., plasma or serum proteins), which may otherwise enhance immune cell recruitment.

It would have also been interesting to better document the recruitment of mpeg1.1‐positive cells following RBC injection at 1 dpi under control and glycated conditions. Based on the observations at 2 dpi (increase in the phagocytic process), we can speculate that glycation of RBCs may have enhanced the early interaction (binding activity) between immune cells and injected RBCs during the first day post‐injection, but this timing would probably be too early to clearly observe the phagocytosis process.

Finally, although the use of autologous RBCs would have been preferable, especially considering the immune system and also the nucleated nature of zebrafish erythrocytes, this approach remains challenging due to the limited control over coagulation processes and the osmotic conditions required to prevent RBC lysis.

### 7.5 Further Perspectives and Conclusions

Interestingly, our work shows that glycation of RBC enhances erythrophagocytosis in a model of ICH in zebrafish. Such an effect is clearly independent of the metabolic disruption of the brain parenchyma itself, given that we carried out our study in normo‐glycemic, non‐diabetic fish. Indeed, we can assume that during hyperglycemia and glycation processes (i.e., diabetes), the hyperglycemic environment can impair brain response by disrupting macrophages, microglia, and other cerebral components' function in ICH. Our model nevertheless allows us to better understand the specific role of RBC glycation on erythrophagocytosis in the brain. However, in diabetic or chronic hyperglycemic conditions, microglia, neurons, astrocytes, and also macrophages are subjected to glycation that may impair their functions. It is therefore important to consider such aspects in the real processes of erythrocyte clearance and regeneration after ICH.

In order to study these aspects, we could use established models of diabetes and diet‐induced obesity (Capiotti et al. 2014; Dorsemans et al. 2017; Ghaddar, et al. 2021) to better understand the real impact of metabolic impairment on brain erythrophagocytosis. For that, we would take advantage of double transgenic fish *Tg(gata1:Red)x(mpeg1.1:GFP)* line allowing us to label zebrafish erythrocytes and microglia/macrophages, respectively.

Moreover, appropriate microgliosis is necessary for efficient brain repair (Tschoe et al. 2020; Guo et al. 2022; Kyritsis et al. 2012; Palsamy et al. 2023). Our experiments conclude that RBC glycation leads to enhanced erythrophagocytosis by microglia/macrophage cells. Further analysis is needed to understand this fully, especially looking at neuroinflammation and if accelerated RBC phagocytosis affects the survival of immune cells and regeneration.

In conclusion, we developed a simplified model to study the consequences of ICH in adult zebrafish. During brain damage, RBC glycation did not influence immune cell recruitment but led to an increase of erythrophagocytosis. Increased erythrophagocytosis of glycated RBCs could then favor microglia and macrophage dysfunction, in particular iron overload, which could induce ferroptosis and consequently result in more severe brain damage.

## Author Contributions

Conceptualization: E.M., P.R., O.M., N.D., D.C. Formal analysis: E.M., P.R., N.D. Investigation: E.M., P.R., N.D. Methodology: all authors. Validation: E.M., P.R., N.D., D.C. Visualization: E.M., P.R., N.D. Writing‐original draft: E.M., P.R., N.D., D.C. Writing‐review and editing: all authors. Project administration: P.R., N.D., D.C., O.M. Supervision: N.D., D.C., P.R. Funding acquisition: D.C., O.M.

## Funding

This study is partly funded by the European Regional Development Fund (CoBRA [EU‐Région Réunion‐French State national counterpart], RE00227771) and the University of Reunion Island. Funding was also obtained from the CHU Réunion Sud, the DéTROI laboratory, Inserm, and the University of Reunion Island.

## Conflicts of Interest

The authors declare no conflicts of interest.

## Supporting information


**Data S1:** cph470088‐sup‐0001‐DataS1.docx.

## Data Availability

The data that support the findings of this study are available from the corresponding author upon reasonable request.
