# Supplementary material for: Red Blood Cell Glycation Triggers In Vivo Cerebral Erythrophagocytosis in Adult Zebrafish in a Model Mimicking Hemorrhagic Stroke
Source: Compr Physiol. 2026 Jan 6;16(1):e70088. doi: 10.1002/cph4.70088 (PMC12771552; doi:10.1002/cph4.70088)
Supplement: Supplementary file 1 — Data S1: cph470088‐sup‐0001‐DataS1.docx. [file CPH4-16-e70088-s001.docx]

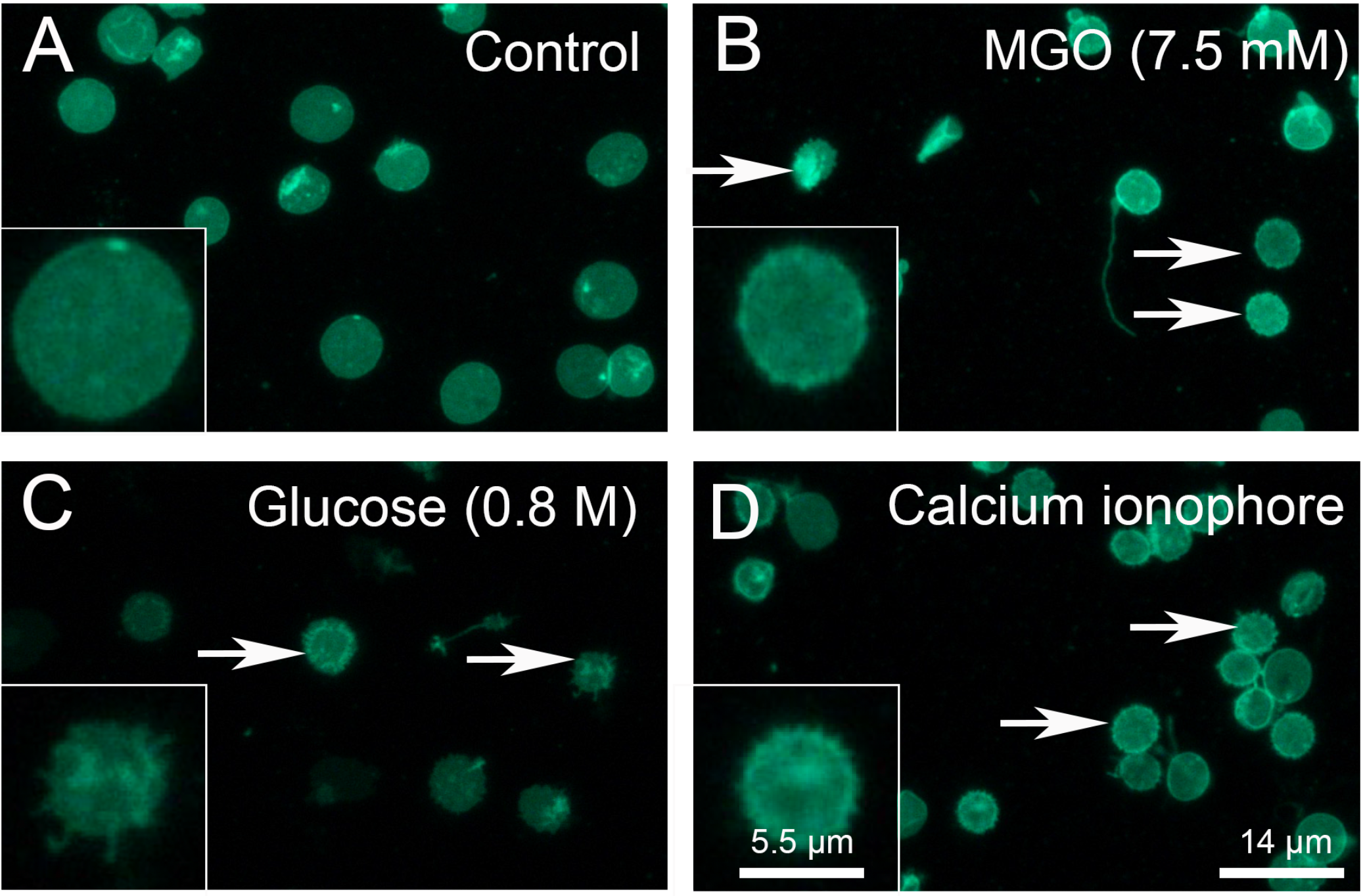


***Suppl. Figure 1: RBC glycation induces morphology defects characteristic of senescent erythrocytes***

Representative pictures of control PKH67-labeled **(A)**, MGO-glycated **(B)** and Glucose-treated **(C)** RBCs showing an impaired membrane morphology harboring showing micro-bleb and spike-like structures, similar to the changes induced by calcium ionophore **(D)**. Arrows indicate examples of such impairments. Scale bar: 5.5 and 14 µm.

***
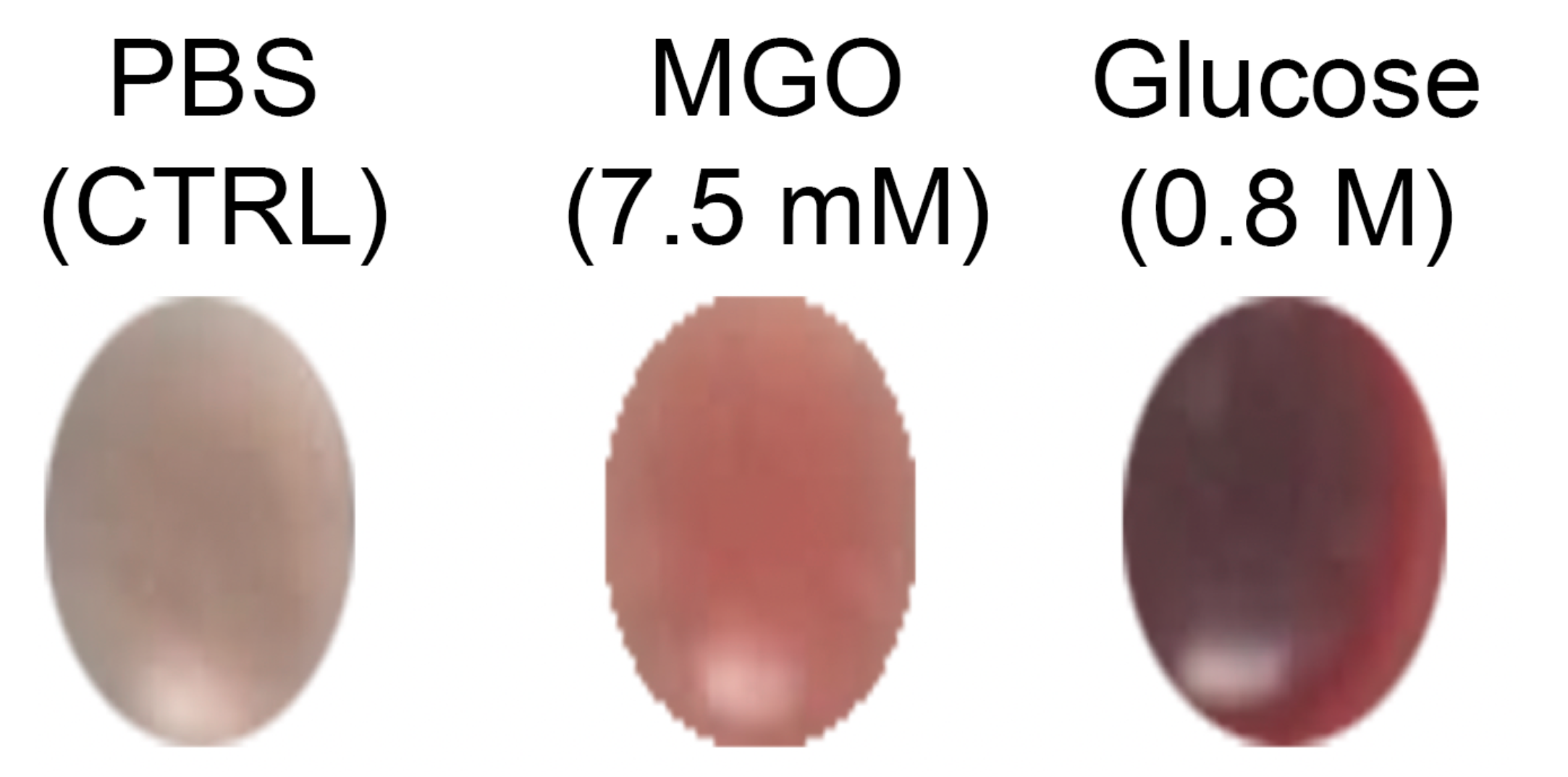
***

***Suppl. Figure 2: Glycation weakens erythrocytes and promotes hemolysis***

Representative images of the supernatant deposited in a 96-well plate after incubation with PBS (CTRL), 7.5 mM MGO (24h) and 0.8 M glucose (3h) in comparison with the control incubation. Note that MGO and Glucose favors hemolysis.


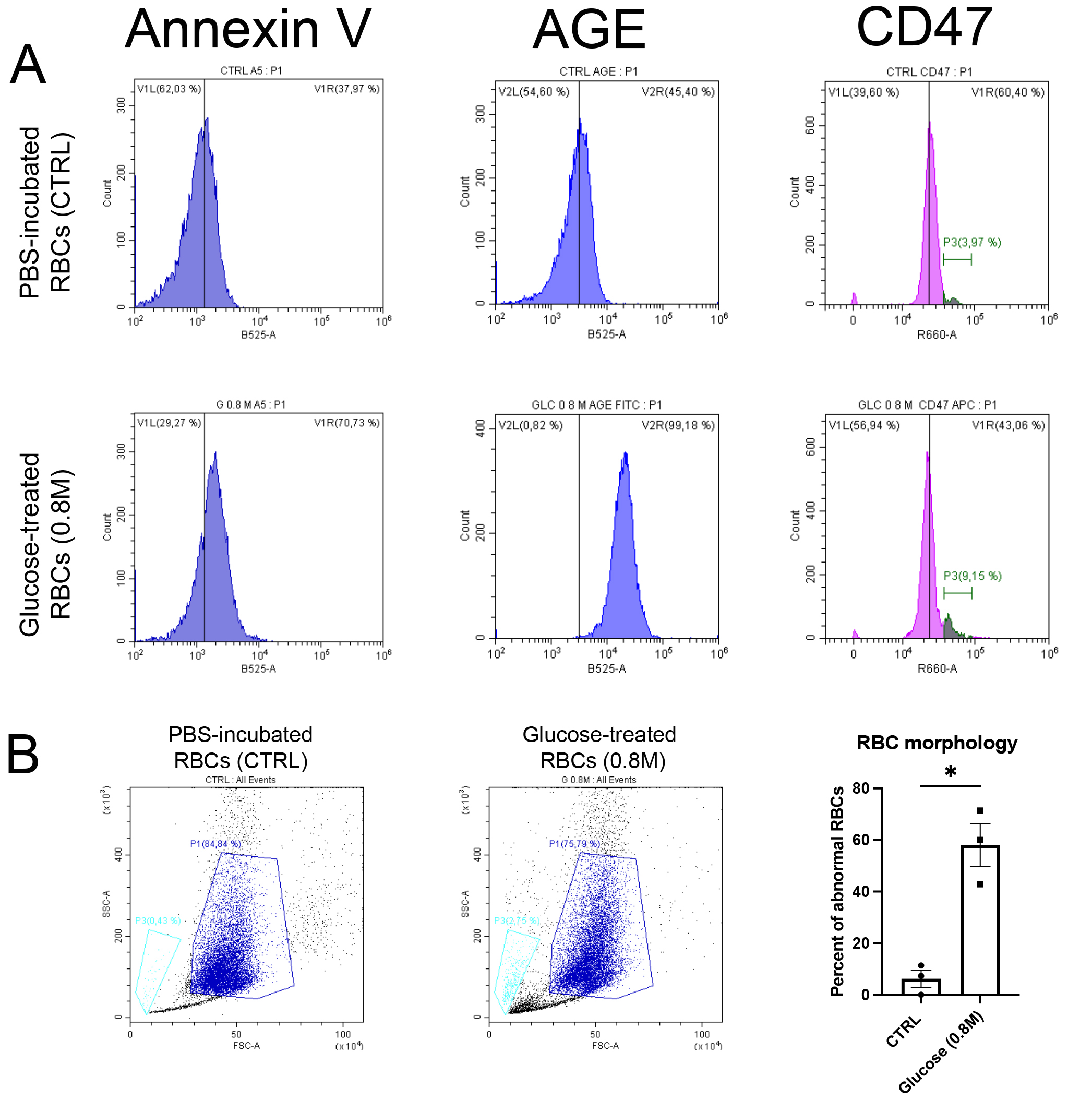


***Suppl. Figure 3: Supraphysiological glucose incubation leads to RBC glycation, favoring eryptosis and morphological defects***

**(A)** Representative flow cytometry diagrams of Annexin V-FITC, AGE and CD47 fluorescence intensity in CTRL and glucose-treated (0.8M) conditions. **(B)** Representative scatter plots with intact RBCs (dark blue) and debris (light blue) which are increased in glycated conditions indicating more RBC damage. The last graph shows a quantification of abnormal RBCs from one experiment resulting from the analysis of three blood samples.
